# Health, Happiness and Eating Together: What Can a Large Thai Cohort Study Tell Us?

**DOI:** 10.5539/gjhs.v7n4p270

**Published:** 2015-01-14

**Authors:** Vasoontara Yiengprugsawan, Cathy Banwell, Wakako Takeda, Jane Dixon, Sam-ang Seubsman, Adrian C Sleigh

**Affiliations:** 1 National Centre for Epidemiology and Population Health, Research School of Population Health, The Australian National University, Canberra, Australia; 2 School of Human Ecology, Sukhothai Thammathirat Open University, Nonthaburi, Thailand

**Keywords:** eating alone, solo eating, commensalism, unhappiness, cohort study, Thailand

## Abstract

Our research investigates the significance of frequent solo consumption of main meals and the association with a holistic wellbeing measure of happiness using data from 39820 Thai Cohort Study members who completed 8-year follow-up in 2013. This nationwide cohort has been under study since 2005 to analyse the dynamics and determinants of the health-risk transition from infectious to chronic diseases. Here we analyse data from the 2009 and 2013 follow-ups.

Approximately 11% reported eating more than half of the main meals per week alone. Sociodemographic attributes associated with eating alone were being male, older age, unmarried, smaller household, lower income, and urban residence. Dissatisfaction with amount of spare time (ie ‘busyness’) was also linked to eating alone. In the multivariate cross-sectional model, reporting being unhappy was associated with frequent solo eating (Adjusted Odds Ratio – AOR 1.54, 95% Confidence Intervals 1.30-1.83). Stratified by age and sex groups, the effects were strongest among females (AOR 1.90 1.52-2.38). A monotonic relationship linked frequent eating alone and 4-year longitudinal unhappiness. The larger the dose of unhappiness the greater the odds of eating alone – AOR 1.29, 1.31, 1.72 after controlling for potential covariates.

Having a meal is not only important for nutritional and health outcomes; it is also a vital part of daily social interaction. Our study provided empirical evidence from a non-Western setting that sharing meals could contribute to increasing happiness.

## 1. Introduction

Feasting with family and friends, and even strangers, as a way of building connections, cementing ties and experiencing nurture is shared among many cultures (Fischler, 2011; Sobal & Nelson, 2003; Young et al., 2009). Both everyday commensality and special feasts are often associated with happiness (Sidenvall et al., 2000). With globalization, commensal rites like modern Christmas feasts have even spread further across classes and nations, and become a universal cultural icon of the ideal family (Gillis, 1989; Pitts et al., 2007). Happiness of individuals is closely linked with happiness of others in the individual’s social network (Fowler & Christakis, 2008). Therefore, a set of good social relationships, including relationships over a meal, is one of the indicators of an individual’s happiness. But is this always the case or does the converse sometimes apply? Are people who usually eat alone more likely to be unhappy or are unhappy people more likely to dine alone?

These are not frivolous questions. Globally, increasing numbers of people now live alone (Japanese Ministry of Health Labor and Welfare, 2012; US Census Bureau, 2011). As populations age more elderly people are living alone while increasing work demands and social mobility pressures mean that younger people are delaying families and are often located in cities and countries far removed from their families. These demographic and lifestyle changes contribute to people having fewer opportunities to dine with others. Overall, the number of meals that people share is decreasing (Mestdag & Glorieux, 2009). Worldwide depression and poor mental health are on the rise (Ferrari et al., 2013; Lepine & Briley, 2011) so the possible contribution of eating alone is important to investigate in a variety of cultures.

Research shows that social context, particularly the presence of others or not during mealtime, has a greater influence on food consumption than the basic physiological functions of hunger and satiety (Herman et al., 2003). For example, eating commensally, or alone, can influence the amounts and types of food consumed via an array of pathways (Bell & Pliner, 2003; Hetherington et al., 2006; Pliner & Bell, 2009). Eating alone may be associated with a lower energy intake which may be positive if weight reduction is a goal but is negative if sociability is the intention. It can also be associated with lose of motivation to eat and some eating disorders like anorexia and binge eating (Donini et al., 2003; Nasser et al., 2004).

Relatively little is known on this topic in the general population although some research on food intake has been conducted in experimental settings (Hetherington et al., 2006), or with vulnerable or special groups, such as children, adolescents and the elderly (Eisenberg et al., 2004; Kimura et al., 2012; Larson et al., 2007). In our large observational study, we examine the relationships between eating commensally or alone and happiness using data from a large cohort of Thai adults. Commensalism is an important cultural ritual among Thais (Seubsman et al., 2009; Yasmeen, 2000) but Thai culinary practices are changing under the growing influence of a westernised food culture which may also include changes to ways of dining as well as changes to the diet. Our research investigates the significance of frequent solo consumption of main meals and the association with a holistic wellbeing measure of happiness.

## 2. Methods

### 2.1 Study Design and Setting

This study uses data from the Thai Cohort Study set up a decade ago to investigate the health-risk transition from infectious to chronic diseases. The Study began with a 20-page mail based questionnaire sent to distance learning adults who were enrolled in the Sukhothai Thammathirat Open University but residing countrywide in 2005 (Sleigh et al., 2008). Out of approximately 200,000 adult students, 87151 responded at baseline. There have been two follow ups since 2009 (n=60569) and again in 2013 (n=39820). The self-administered questionnaires include a wide range of information including sociodemographic characteristics, health behaviours (smoking, drinking, physical activity), doctor diagnosed conditions, weight and height, eating habits and food choice, physical and mental health, and personal wellbeing including life satisfaction and happiness. For the analyses of happiness and commensalism presented here, we used the 2009 and 2013 follow-ups because they included information on the key variables of interest.

### 2.2 Exposure, Outcome, and Potential Covariates

The exposure of interest–eating main meals alone–was measured in the 2013 follow-up questionnaire. In a section on food there was a question on eating alone: “In the past 7 days, how many times did you eat a meal alone?” Those reporting 4+ times per week were categorised as ‘frequent eating alone’; the ‘main meal’ measure is equivalent to number of days per week. Two other related questions include “how many times have you eaten per day (including main meals and snacks)” and “whether when you eat alone, do you eat more, less or the same as when you eat with others?” The outcome is measured in a separate section related to personal wellbeing where one question in standard format asks: “In the past 4 weeks, how much of the time did you feel happy?” Possible responses were all, most, some, a little, or none of the time. The last two categories ‘little’ or ‘none’ of the time were combined as being ‘unhappy.’

Potential covariates in this study include: sex, age, marital status, family size, household monthly income, occupation, work hours per week, and current residence. We also include two health-related covariates which included the first question of the international standard self-rated overall health (categorised into three groups: excellent/very good/good; fair; poor or very poor) and body mass index categories (standard calculation based on self-reported weight and height). Asian cut-offs were based on International Obesity Task Force categories for Asians (Kanazawa et al., 2002): which included: underweight (BMI<18.5), normal (18.5 to <23), overweight (23 to <25), obese I (25 to <30), and obese II (BMI≥30). One social covariate asks: “Thinking about your own life and personal circumstances, how satisfied are you with the amount of spare time you have?” Note that this is a proxy for ‘busyness’. Responses ranged from 0 (completely dissatisfied) to 10 (completely satisfied): cohort members who rated 0-3 were categorised as ‘not satisfied’, 4-8 as ‘somewhat satisfied’, and 9-10 as ‘very satisfied’.

For a longitudinal analysis, we were directionally constrained by lack of longitudinal data on eating alone. However, we did have 4-year longitudinal data on happiness allowing us to categorise cohort members by their ‘longitudinal happiness’ (2009-2013) and investigate association with eating alone in 2013 (ie happiness as exposure, eating alone as outcome).

### 2.3 Statistical Analyses

Descriptive analyses include distribution of unhappiness and frequent eating alone by cohort characteristics, followed by age-sex adjusted bivariate Odds Ratios and 95% Confidence Intervals. Individuals with missing data for given analyses were excluded, thus totals could vary due to available information. However, missing data usually only reflected less than 5 percent of observations. Given the large size of the dataset, results were unlikely to be effected by missing data. For subsequent analyses, multivariate regression was used to estimate association between unhappiness and frequency categories of eating alone per week, after adjusting for potential covariates. The results were reported by adjusted odds ratios and 95% confidence intervals.

### 2.4 Ethical Approval

Informed written consent was obtained from all participants. Ethics approval was obtained from Sukhothai Thammathirat Open University Research and Development Institute (protocol 0522/10) and the Australian National University Human Research Ethics Committee (protocols 2004/344 and 2009/570). All students were assured that their information was confidential and their identity would never be revealed. Data were anonymized before analysis.

## 3. Results

Among cohort members in 2013, 45.3% were males, 45.4% aged less than <35 years and 44.0% aged between 35-49 years, and approximately two thirds were married (Table 1). About 40% were professionals and 30% were office assistants. Unhappiness (happy ‘little’ or ‘none of the time’) was reported by 5.4% of cohort members. Age-sex adjusted bivariate analyses have shown that older cohort members (50+ years), being divorced-separated-widowed, or having low household monthly income were all associated with being unhappy. Poor self-assessed health, having abnormal body size (underweight or obese), and dissatisfaction with amount of spare time were also associated with reported unhappiness.

**Table 1:** Distribution and bivariate association of frequent eating alone and unhappiness, Thai Cohort Study 2013

Cohort attributes	Overall (column % n=39820)	Frequent eating alone*		Unhappiness**	

		Distribution (row %)***	Bivariate age-sex adjusted Odds Ratios [95% Conf Intervals]	Distribution (row %)***	Bivariate age-sex adjusted Odds Ratios [95% Conf Intervals]
**Overall**		11.1		5.4	
**Age and sex categories:**					
Males	45.3	11.7	Reference	5.6	Reference
Females	54.6	10.6	**0.71** [0.66-0.76]	5.3	0.96 [0.88-1.06]
<35 years	45.4	10.8	Reference	2.1	Reference
35-49 years	44.0	11.3	1.07 [0.99-1.17]	4.2	**2.08** [1.88-2.31]
50+ years	10.5	11.2	**1.25** [1.12-1.40]	5.1	**2.55** [2.22-2.94]
**People in the household:**					
1-2	20.3	12.7	Reference	5.2	Reference
3-4	47.8	10.8	**0.74** [0.70-0.79]	5.5	1.05 [0.93-1.18]
5+	31.8	10.5	**0.72** [0.67-0.76]	5.3	1.02 [0.89-1.17]
**Marital status:**					
Married	62.4	10.4	Reference	4.8	Reference
Not married	29.3	12.1	**1.48** [1.36-1.61]	7.9	**1.27** [1.15-1.42]
Divorced/separated/widowed	8.2	12.6	**1.53** [1.34-1.74]	5.9	**1.74** [1.51-2.02]
**Household monthly income:**					
<10000 Baht	12.5	11.1	**1.61** [1.50-1.74]	8.5	**2.08** [1.83-2.37]
10001-20000 Baht	18.4	11.9	**1.40** [1.32-1.50]	6.0	**1.45** [1.28-1.65]
20001-30000 Baht	23.4	10.9	**1.20** [1.14-1.28]	5.3	**1.24** [1.10-1.41]
>30000 Baht	25.7	10.8	Reference	4.3	Reference
**Occupation:**					
Professionals/managers	41.7	10.6	Reference	5.0	Reference
Office assistants	31.6	11.7	**1.18** [1.08-1.28]	5.1	1.01 [0.90-1.13]
Skilled/manual workers	19.2	12.1	**1.24** [1.12-1.37]	5.9	**1.17** [1.03-1.33]
Others/not applicable	7.5	9.5	1.05 [0.90-1.23]	7.4	**1.54** [1.30-1.81]
**Work hours per week:**					
0-20	7.4	11.0	Reference	6.6	Reference
21-40	37.3	10.6	0.78 [0.67-0.91]	4.7	**0.68** [0.57-0.82]
40+	48.3	11.8	0.94 [0.81-1.09]	5.2	**0.76** [0.64-0.90]
**Current residence:**					
Rural	44.9	10.6	Reference	5.4	Reference
Urban	55.0	11.5	**1.12** [1.04-1.20]	5.4	1.00 [0.92-1.10]
**Region:**					
Bangkok	15.6	11.8	**1.14** [1.03-1.25]	5.3	1.03 [0.97- 1.17]
Non-Bangkok	84.4	11.0	Reference	5.5	Reference
**Self-rated health:**					
Excellent/very good/good	66.6	10.5	Reference	3.9	Reference
Fair	27.8	12.3	**1.32** [1.22-1.43]	7.0	**1.85** [1.67-2.04]
Poor or very poor	5.6	12.5	**1.46** [1.26-1.71]	15.1	**4.45** [3.89-5.11]
**Body mass index categories:**					
Underweight (≤18.5)	5.8	10.3	1.01 [0.91-1.11]	6.6	**1.34** [1.11-1.62]
Normal (18.5 to <23)	43.7	10.6	Reference	5.0	Reference
Overweight (23 to <25)	21.8	11.4	1.04 [0.98-1.04]	5.1	1.00 [0.89-1.14]
Obese I (25 to <30)	23.4	11.4	1.06 [1.00-1.12]	5.8	**1.16** [1.04-1.32]
Obese II (≥30)	5.3	13.4	1.06 [0.95-1.18]	6.8	**1.36** [1.13-1.66]
**Satisfaction with spare time:**					
Not satisfied	8.2	12.5	**1.29** [1.12-1.47]	14.2	**3.63** [3.16-4.16]
Somewhat satisfied	62.6	11.1	1.04 [0.96-1.13]	4.7	1.09 [0.97-1.21]
Very satisfied	29.2	10.9	Reference		Reference

* Cohort members were asked: “In the past 7 days, how many times did you eat a main meal alone?” Those reporting 4+ times per week were categorised as ‘frequent eating alone’.

** Cohort members were asked: “in the past 4 weeks, how much of the time did you feel…happy?” Possible responses were all, most, some, a little, or none of the time. Last two categories ‘little or none of the time’ were combined as ‘unhappiness’.

*** Row percent shows the prevalence of frequent eating alone or unhappiness within each category of cohort attributes.

There were 11.1% of cohort members who reported eating more than half of the main meals per week alone. Attributes associated with frequent eating alone include being male; older age, being divorced-separated-widowed, having a small household, having lower income, being an office assistant or a skilled manual worker (compared to a professional or a manager), and residing in urban areas. Poor self-assessed health, but not abnormal body size, and dissatisfaction with spare time were associated with frequent eating alone. We have also investigated the effect of eating alone on meal size (data not shown) and found about 8% reported eating more, 32% less, and 60% about the same.

We reported our multivariate analyses based on logistic regression (Table 2), adjusting for potential covariates in Table 1 by overall, sex, and age groups. In the multivariate cross-sectional model, reporting being unhappy was associated with frequent solo eating (Adjusted Odds Ratio – AOR 1.54, 95% Confidence Intervals 1.30-1.83). Stratified by age and sex groups, the effects were strongest among females (AOR 1.90 1.52-2.38).

**Table 2:** Associations between frequency of unhappiness and eating main meals alone, Thai Cohort Study 2013

Happiness status in 2013		Multivariate adjusted Odd Ratios of frequent eating alone** [95% Confidence Intervals]				

		Overall	Male	Female	<40 years	40+ years
All or most	60.0	Reference	Reference	Reference	Reference	Reference
Some of the time	34.6	1.34 [1.23-1.46]	1.25 [1.15-1.36]	1.29 [1.15-1.43]	1.29 [1.16-1.44]	1.40 [1.23-1.59]
Little/none	5.4	1.54 [1.30-1.83]	1.35 [1.14-1.61]	1.90 [1.52-2.38]	1.54 [1.23-1.94]	1.53 [1.17-1.99]

*Frequent eating alone was measured by cohort members who reported eating alone four times or more per week; as the ‘main meal’ measure is equivalent to days per week;

** Adjusted for all covariates in Table 1.

We also found a longitudinal association between 2009-2013 unhappiness and frequent eating main meals alone (Table 3). About 90% of cohort members reported being happy ‘some’, ‘most’, or ‘all’ of the time in both waves and approximately 8% reported unhappiness either in 2009 or 2013. Only 1% reported being unhappy in both waves. A monotonic relationship linked frequent eating alone and 4-year longitudinal unhappiness. The larger the dose of unhappiness the greater the odds of eating alone – AOR 1.29, 1.31, 1.72 after controlling for potential covariates.

**Table 3:** Longitudinal unhappiness and frequency of eating main meals alone, Thai Cohort Study 2009-13

Longitudinal happiness status 2009 and 2013 (column %)	Happiness status 2009-13 (column %)	Eating alone 4+ times per week* (row %)	Multivariate adjusted Odd Ratios [95% Confidence Interval]**
2009 + 2013 + (unhappy neither years)	90.8	11.0	Reference
2009 - 2013 + (unhappy in 2005)	4.4	12.3	1.29 [1.08-1.54]
2009 + 2013 - (unhappy in 2009)	3.7	12.9	1.31 [1.09-1.57]
2009 - 2013 - (unhappy both years)	1.0	14.1	1.72 [1.20-2.46]

* For the ‘main daily meal’;

** Adjusted for all covariates in Table 1; self-reported unhappiness was measured by cohort members who reported being happy ‘little’ or ‘none’ of the time in 2009 or in 2013.

## 4. Discussion

We investigated the relationship between frequent eating alone and unhappiness in a large sample of middle and older age Thais. We found a strong significant association, particularly among females and cohort members aged 35 years and younger. This association was cross-sectional; notably, there was a mutual dose-response whereby an increased level of one variable connected to an increased level of the other. Our longitudinal results have shown that frequently eating main meals alone is associated with unhappiness, after adjusting for potential covariates. As well, longitudinal analyses confirmed that 4-year unhappiness status is also strongly associated with frequent eating main meals alone at the end of the study period.

Our study contributes to the current literature on eating alone (Hetherington et al., 2006; Pliner & Bell, 2009) by providing epidemiological evidence on non-Western middle-aged working adults. Public eating in Thailand is commonplace (whether food stalls, restaurants or shopping malls), and Thais consider it “bad luck” to dine alone in this situation. At home, Thais are said to “value the idea that family should eat together” (Walker, 1996) although it is not a rigid tradition and not everyone in the household may eat at the same time (Seubsman et al., 2009; Yasmeen, 2000). Our study has shown adverse psychological effects of frequent eating main meals alone, which may reflect the cultural norm of eating with others or reflect an existing psychological state.

Our research strength is uniquely inclusive of a wide array of health and social covariates within a very large national sample of Thai adults who represent well geographic residence and average income for the general Thai population (Sleigh et al., 2008). In previous analyses of these Thai cohort data, happiness correlated with other positive wellbeing measures (Yiengprugsawan et al., 2012) and was predictive of lower all-cause mortality (Yiengprugsawan et al., 2014).

We also note the possible limitations of the study including the nature of self-reported happiness which could be subject to cultural constraint. Our study provided evidence of both cross-sectional and 4-year associations between unhappiness and eating alone; however, since our study did not have longitudinal information on eating alone status, we were not able to make conclusions on the directions of causal inference. Recent literature has shown that eating alone could be a major behavioural symptom of binge eating disorders due to embarrassment of eating in front of others (White & Grilo, 2011). Also stress from communal eating for some could lead to eating alone (Danesi, 2012). Further research on this area could investigate possible bidirectional causation providing insight into the complex relationship between psychological health and eating status.

Eating with others is thought to be an entrenched behaviour that has assisted humans to survive as a species (Lee, 2008) but we do not have information on the mechanisms linking frequent eating alone and unhappiness. Plausible explanations could be due to a modern busy lifestyle (as shown in our ‘busyness’ proxy data related to dissatisfaction with the amount of spare time). As well this could potentially be due to loneliness, a subjective experience of social isolation with serious impacts on cognition, emotion, behaviour, and health (Bofill, 2004; Hawkley & Cacioppo, 2010). Our results could stimulate similar studies in other parts of the world. Future research could also address associations in non-Western culture between solo or commensal dining on other health-related outcomes.

## 5. Conclusion

Consuming food is a vital part of daily social interaction and the reverse eating alone can be seen as a strong experience of loneliness. Shared family meals and happiness have significance in special annual celebrations; as well, happiness can also be manifested in daily life through more frequent sharing meals with others. This applies in Buddhist Asia as well as Christian societies elsewhere and so hints at commensalism being valued as a universal cross-cultural human trait. Rapid changes of lifestyles and food environment now make eating commensally more difficult, or even challenging many societies including Thailand. At the same time, people long to eat together to promote happiness and a sociable life.
